# Contrasting Effects of Grass-Derived Endophytic Fungal VOCs on Early Growth of Spring Barley and Red Clover: From Stimulation to Suppression

**DOI:** 10.3390/microorganisms14030533

**Published:** 2026-02-25

**Authors:** Izolda Pašakinskienė, Saulė Matijošiūtė, Violeta Stakelienė, Marius Rimkevičius, Jurga Būdienė

**Affiliations:** 1 Botanical Garden, Vilnius University, Kairėnų 43, 10239 Vilnius, Lithuania; 2 Department of Organic Chemistry, State Research Institute Centre for Physical Science and Technology, Saulėtekio av. 3, 10257 Vilnius, Lithuania; jurga.budiene@ftmc.lt

**Keywords:** endophytic fungi, volatile organic compounds (VOCs), *Cadophora fastigiata*, plant growth promotion, sesquiterpenes, plate-in-plate assays

## Abstract

Endophytic fungi can influence plant development through diverse molecular mechanisms; however, their volatile organic compound VOC-mediated effects on agriculturally relevant crops remain insufficiently characterized. In this study, we examined the effects of VOCs produced by six grass-root-associated endophytic fungi—*Cadophora fastigiata*, *Cordyceps fumosorosea*, *Chaetomium funicola*, *Epicoccum nigrum*, *Microdochium bolleyi*, and *Plectosphaerella cucumerina*—on early growth of spring barley (*Hordeum vulgare*) and red clover (*Trifolium pratense*). In plate-in-plate VOC exposure assays, we assessed root system traits, root hair formation, and biomass accumulation. Responses to fungal VOCs were fungal species-specific but similar across barley and red clover. VOCs emitted by *C. fastigiata* and *P. cucumerina* were consistently associated with increased root growth, root hair proliferation, and seedling biomass, whereas VOCs from *M. bolleyi* and *C. funicola* resulted in neutral or growth-suppressing effects. A complementary seed inoculation experiment was conducted with barley, which showed fungal species–dependent contrasting effects consistent with the observations of VOCs treatment. Gas chromatography–mass spectrometry (GC–MS) analysis revealed that *C. fastigiata*, the isolate associated with the strongest growth-promoting responses, emitted a diverse VOC profile dominated by sesquiterpenes, with 22 compounds identified. Together, these results demonstrate that VOCs emitted by grass-root-associated endophytic fungi exert reproducible, species-specific effects on early plant development occurring in phylogenetically distant species. The findings highlight the value of VOC-based assays for comparative functional screening of fungal isolates, providing a foundation for future studies that aim to link individual VOCs to plant growth responses.

## 1. Introduction

Endophytic fungi play a crucial role in shaping plant performance through a diverse range of biochemical and molecular interactions [1,2,3,4]. These fungi inhabit internal plant tissues without causing disease and include lineages that exhibit neutral or mutualistic lifestyles, depending on the host species and environmental context [5,6]. Among the mechanisms by which endophytes affect plant development, volatile organic compounds (VOCs) have emerged as particularly potent signaling molecules capable of modulating plant architecture and physiology [7,8,9,10].

Numerous studies have demonstrated that microbial VOCs are capable of reprogramming plant development [11,12,13,14,15]. VOCs influence plant root architecture, shoot growth, hormone balance, nutrient uptake, and they also participate in cross-kingdom communication. Fungal species exhibit significant diversity in their volatile profiles, with metabolic signatures that vary by strain, lifestyle, ecological niche, and growth conditions [16,17]. While microbial VOC research has expanded rapidly, most work has centered on bacterial [18,19] or ectomycorrhizal fungi VOCs [20]. Fungal endophytes have received comparatively less attention, and their volatile-mediated interactions with agronomically relevant crops remain poorly characterized.

This knowledge gap is particularly relevant for spring barley (*Hordeum vulgare*, Poaceae) and red clover (*Trifolium pratense*, Fabaceae), which are essential components of temperate agroecosystems and exhibit distinct differences in root morphology, resource acquisition strategies, and microbial association patterns (for barley [21,22]; for red clover [23,24]). Barley is widely cultivated as a cereal crop, and its early vigor, root development, and nutrient uptake critically influence grain yield [25,26]. Red clover, a key forage legume, provides high-protein biomass, supports nitrogen fixation, and contributes to soil fertility and pasture quality [27]. Although microbial inoculation has been applied to cereals and legumes [28,29,30], the potential of fungal VOCs as growth-modifying agents in spring barley and red clover has been underexplored. Studying these two contrasting crops allows evaluation of whether plant responses are conserved across phylogenetically and physiologically distant crops.

Our previous work has demonstrated that inoculation with *Cadophora fastigiata*, *Paraphoma fimeti*, and *Plectosphaerella cucumerina* promotes growth in spring barley and Italian ryegrass [31]. Similarly, *Epicoccum nigrum* in sugarcane [32] and *Cordyceps fumosorosea* in eggplant [33] have been shown to enhance root and shoot development. In contrast, other grass-root-associated fungi, such as *Microdochium bolleyi*, which is widely common in root associations across grasses [34], are known to function as neutral or weakly antagonistic symbionts [5,35]. Together, these studies highlight substantial functional variation among grass-root-associated endophytes.

Based on this background, the six endophytic fungi used in the present study—*Cadophora fastigiata*, *Cordyceps fumosorosea*, *Chaetomium funicola*, *Epicoccum nigrum*, *Microdochium bolleyi*, and *Plectosphaerella cucumerina*—were selected from our laboratory culture collection [34,36] according to the following criteria: (i) their frequent occurrence in grassland root systems, (ii) contrasting functional traits reported in previous studies, and (iii) their taxonomic diversity. To our knowledge, the VOC-mediated developmental responses of these fungi have not been systematically evaluated across cereal and forage legume crops, nor have they been linked to their VOC profiles.

To address this gap, we employed an integrated experimental framework. This included combining in vitro VOC exposure assays to assess early development of spring barley and red clover, a greenhouse experiment to evaluate barley growth responses to fungal inoculation, and gas chromatography–mass spectrometry (GC–MS) to characterize the VOC profiles of the most growth-promoting fungal isolates.

Although initial experiments evaluating the impact of VOC exposure on *C. fastigiata* and *P. cucumerina* in barley have been reported [31], in this study, we extended the experiment to six fungal species and evaluated additional seedling growth parameters in two contrasting crop plants, providing a broader analysis of VOC effects.

## 2. Materials and Methods

### 2.1. Plant Material and Fungal Isolates

Seeds of the spring barley variety ‘Gunda DS’ and the red clover variety ‘Arimaičiai’ were obtained from the Breeding Department of the Institute of Agriculture, Lithuanian Centre for Agricultural and Forestry Sciences, Akademija, Kėdainiai district, Lithuania.

For VOC exposure in a plate-in-plate experiment, six root endophytic fungal isolates from the Vilnius University Botanical Garden Laboratory collection were used: *Cadophora fastigiata* isolate BSG003 and *Chaetomium funicola* BSG039 isolated from the roots of *Festuca gigantea*; *Plectosphaerella cucumerina* BSG006 and *Microdochium bolleyi* BSG007 from *Lolium perenne* x *Festuca gigantea* hybrids (laboratory plant collection), *Epicoccum nigrum* BSG025 from *Lolium multiflorum*, and *Cordyceps fumosorosea* BSG015 from *Lolium temulentum.* Fungal strain isolation procedures and a taxonomic assignment were performed as described by Stakelienė et al. [34] and Pašakinskienė et al. [36].

### 2.2. Spring Barley and Red Clover Seed Sterilization

Barley ‘Gunda DS’ seeds for all fungal VOC treatments and controls were surface-sterilized following the laboratory protocol: seeds were placed in 50 mL of 1% AgNO_3_ (silver nitrate) solution and agitated on a rotary shaker at 110 rpm for 10 min at room temperature (RT). Seeds were then rinsed three times with sterile water, with the final wash lasting 10 min.

Red clover seeds ‘Arimaičiai’ were surface-sterilized as follows: seeds were treated with 70% ethanol for 1 min, rinsed with sterile water, and then incubated in 50 mL of 2.5% sodium hypochlorite (1:2 dilution of 5% stock; Thermo Fisher Scientific Baltics, Cat. No. P005-03, Vilnius, Lithuania) on a rotary shaker at 110 rpm for 10 min at RT. Seeds were then rinsed three times with sterile water, with the final wash lasting 10 min.

### 2.3. Exposure of Spring Barley and Red Clover Seedlings to Fungal VOCs in Plate-in-Plate Assays

The cultures of the endophytic fungi were grown in 35 mm diameter Petri dishes on PDA medium supplemented with ampicillin (100 µg/mL) and streptomycin sulfate (100 µg/mL) at 27 °C in the dark for 2 days. Two small dishes (without lids) containing the fungus cultures were placed in a large, 120 × 120 mm, square Petri dish filled with ½-strength Murashige and Skoog medium (MS) (the same formulation used for barley and red clover, without additional sucrose or vitamins). Sterilized barley and red clover seeds were placed on the surface of the MS medium in large dishes, which were sealed with Parafilm. This setup allowed the growing plant seedlings to be exposed to VOCs emitted by the fungal mycelium without direct contact. Plate-in-plate assays were positioned on dish dryer racks at a 60° angle and maintained at 24–26 °C under a 16 h photoperiod, with artificial light at 120 µmol·m^−2^·s^−1^ (BIOLUX, L 36W/965, OSRAM, Regensburg, Germany).

For spring barley, three experimental series were conducted separately in time, each with its own control containing no fungal colonies in the small Petri dishes. Series 1 included assays with *E. nigrum* and *M. bolleyi*; Series 2 included *C. fastigiata* and *P. cucumerina*; and Series 3 included *C. fumosorosea* and *C. funicola*. Four barley seeds were placed in each of 10 square Petri dishes per treatment, including the control (*n* = 40 seeds per treatment).

In the red clover VOC treatment experiment, the same six fungal VOCs were tested. All procedures for setting up the plate-in-plate assays were identical to those used for barley, except that this experiment was conducted as a single series and included one control. Ten red clover seeds were placed in each of five square Petri dishes per treatment, including the control (*n* = 50 seeds per treatment).

### 2.4. Measurements of Plant Growth Parameters

In the barley VOC treatment experiment, seven parameters were measured: primary and lateral root number on day 7, root hair number on days 5, 6, and 7, and root and shoot biomass on day 12. Root hair estimation in 0.5 mm^2^ was performed using a Euromex NexiusZoom EVO stereomicroscope (Cat. No. NZ.1902-B, Euromex Microscopen, Duiven, The Netherlands) at ×35 magnification, and the ImageFocusAlpha software (version 4). For each treatment and control on days 5, 6, and 7, representative roots were selected from 10 square Petri dishes, resulting in *n* = 10 per group. Root hairs were quantified in the lower section of the root hair zone, ~0.5 mm above the onset of root hair formation. Barley shoot and root biomass were estimated using an ABT220-5DM balance (Kern, Balingen, Baden-Württemberg, Germany) (*n* = 40 plants per treatment).

In the red clover VOC treatment experiment, due to slower growth and the dominance of a single primary root, measurements were limited to three parameters: root length, root hair number, and total seedling biomass. Root length was quantified on day 7 (*n* = 50 per treatment). The number of root hairs was quantified microscopically on day 7 using the same method as for barley, with *n* = 10 roots assessed per treatment and control. Total seedling biomass was estimated on day 12 using an ABT220-5DM balance (*n* = 50 plants per treatment).

### 2.5. Spring Barley Seed Inoculation Experiment

The seeds were surface-sterilized and inoculated with spore suspensions of *C. fumosorosea*, *C. funicola*, and *M. bolleyi*, prepared from 14-day-old PDA cultures, as described in Pašakinskienė et al. [31]. In brief, a total of 150 surface-sterilized seeds per treatment were inoculated by mixing them with fungal spore suspensions and incubated in a rotor shaker (Eppendorf SE, Hamburg, Germany) at 140 rpm and 28 °C for 30 min. After inoculation, the seeds were briefly rinsed with sterile water. For the control, 150 sterilized seeds were kept in sterile water, and all procedures—including rotor shaking and rinsing—were performed in parallel with the incubated seeds. In both the control and inoculation treatments, an excess of seeds (*n* = 150) was initially sampled, taking into account that not all would germinate. The seeds were germinated at 22–24 °C in Petri dishes on sterile filter paper moistened with sterile water and were subsequently used for growth assessment assays and microscopic examination of fungal colonization.

For fungal colonization verification, fifty roots per treatment (*n* = 50) were sampled on day 4, stained with 0.025% (*w*/*v*) Trypan Blue, and examined under a light microscope using the procedure described in Pašakinskienė et al. [36].

For growth assessment, well-germinated barley seedlings (*n* = 54 per treatment) were transferred on day 7 from Petri dishes to multi-cavity trays (28 positions, 6.5 × 6.5 × 6.2 cm) filled with an autoclaved (121 °C, 30 min) mixture of peat and compost soil (1:4, *v*/*v*); detailed soil composition described in Pašakinskienė et al. [31]. Plants were grown in a greenhouse under a 16 h light/8 h dark photoperiod (200–220 µmol·m^−2^·s^−1^) at 24–26 °C during the day and 16–18 °C at night. Plants were watered daily to maintain optimal soil moisture.

After 30 days, aerial parts and roots were harvested (*n* = 54 plants per treatment). Fresh shoots were weighed immediately, and roots were gently washed with water, blotted dry with paper towels, and then weighed. Weighing was performed using an EWJ balance (Kern, Balingen, Baden-Württemberg, Germany).

### 2.6. Sampling and Analysis of the Fungal VOCs

Analysis of volatile organic compounds (VOCs) emitted by fungal mycelium was performed using gas chromatography–mass spectrometry (GC–MS). Five sample types were analyzed: colonies of *C. fastigiata*, *P. cucumerina*, and *P. fimeti*; a mixed sample comprising colonies of all three fungi; and a control consisting of a Petri dish containing PDA medium only. All samples were analyzed in three biological replicates (*n* = 3). Samples were collected on day 8 of culture growth, with 4–5 colonies (1–2 cm in diameter) present in each Petri dish sealed with Parafilm. VOCs released by the fungi were collected for 1 h from the headspace using a PDMS/DVB 65 µm solid-phase microextraction (SPME) fiber (Supelco, Bellefonte, PA, USA). Prior to VOC collection, the SPME fiber was desorbed under the following chromatographic conditions: the initial temperature of 50 °C was held for 1 min, then increased at 10 °C/min to 260 °C and maintained for 15 min. The GC injector temperature was set to 250 °C, and the ion source and transfer line temperatures were 220 °C and 250 °C, respectively. VOCs emitted by the fungal mycelium were collected using the SPME fiber at a constant room temperature of 22 °C. Immediately after the VOC collection, the SPME fiber was transferred to a GC–MS system consisting of a Shimadzu GC-2010 gas chromatograph (GC) coupled with a Shimadzu MS-QP 2010 Plus mass selective detector (MS) (Shimadzu, Kyoto, Japan). The GC was equipped with a non-polar Rxi-5SilMS Integra-Guard column (30 m × 0.25 mm × 0.25 µm; Restek, Bellefonte, PA, USA). The compound separation was performed under the following conditions: the initial temperature of 40 °C was maintained for 2 min, then increased to 240 °C at 7 °C/min, and the final temperature was held isothermally for 4 min. Helium was used as a carrier gas at a flow rate of 1.3 mL/min. Electron ionization spectra were acquired at an electron energy of 70 eV. The injector, interface, and ion source temperatures were set at 240 °C, 250 °C, and 220 °C, respectively. MS analysis was performed in a scanning mode over a mass range of 33–400 *m*/*z*. The VOCs were identified by comparing their mass spectra and retention indices with the NIST Mass Spectral Search Program version 2.0 (National Institute of Standards and Technology, Gaithersburg, MD, USA). Each of the three biological repetitions of the sample type had two technical replicates.

### 2.7. Statistical Analysis

Statistical analysis was performed using STATISTICA^®^ version 7. Data normality was assessed using the Shapiro–Wilk test, and statistical significance was evaluated using Student’s *t*-test. Differences were considered significant at *p* < 0.05, *p* < 0.01, and *p* < 0.001. Graphs were drawn using MS Excel software 2016. To visualize patterns across treatments and traits, a heat map with hierarchical clustering was generated using Euclidean distance and complete linkage. This analysis was used for descriptive comparison to help interpret overall response patterns; no multiple comparison corrections were applied.

## 3. Results

### 3.1. The Effect of Fungi-Emitted VOCs on Spring Barley Growth In Vitro

**Figure 1:**
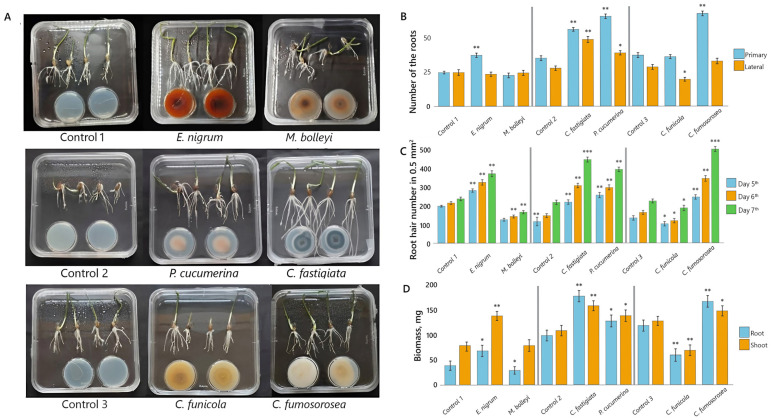
Development of spring barley exposed to VOCs emitted by *E. nigrum*, *M. bolleyi*, *C. fastigiata*, *P. cucumerina*, *C. funicola* and *C. fumosorosea* in plate-in-plate assays (in (**B**–**D**), three experimental series are separated by vertical lines). (**A**) spring barley seedlings after 7 days of growth under exposure to VOCs; (**B**) number of primary and lateral roots per plant after 7 days; (**C**) number of root hairs at 5, 6, and 7 days per 0.5 mm^2^ area; (**D**) fresh root and shoot biomass after 12 days of growth. Notes: the sampling replicates are *n* = 40 in (**B**,**D**), and *n* = 10 in (**C**); the symbols *, **, and *** indicate significant differences relative to control without fungal VOC exposure at *p* < 0.05, *p* < 0.01, and *p* < 0.001, respectively (Student’s *t*-test).

To assess the impact of VOCs emitted by endophytic fungi on spring barley ‘Gunda DS’, six fungal isolates previously obtained from the grass roots were examined. In the first VOC exposure experimental series, *E. nigrum* and *M. bolleyi* were tested; in the second, *C. fastigiata* and *P. cucumerina*; and in the third, *C. fumosorosea* and *C. funicola*, with all treatments compared to controls. In the closed plate-in-plate system, barley seedlings exposed to fungal VOCs showed distinct, species-dependent growth responses compared with non-exposed controls (Figure 1A–D).

Primary and lateral root numbers were recorded on day 7. In four out of six VOC exposure treatments, *P. cucumerina*, *C. fumosorosea*, *C. fastigiata*, and *E. nigrum* increased the number of primary roots by 86%, 73%, 61%, and 50%, respectively (Figure 1B). In contrast, *M. bolleyi* and *C. funicola* had no significant effect.

Lateral root formation was strongly stimulated by *C. fastigiata* and *P. cucumerina*, with increases of 82% and 41%, respectively, compared to the control (Figure 1B). Conversely, *C. funicola* markedly suppressed lateral root development, reducing their number by 35%. In other treatments, including those with *C. fumosorosea*, *E. nigrum*, and *M. bolleyi*, the number of lateral roots remained similar to that of the control.

The effects of fungal VOCs on root hair formation were evaluated on days 5, 6, and 7. Root hair growth varied markedly among treatments (Figure 1C). By day 7, exposure to VOCs from *C. fumosorosea*, *C. fastigiata*, *P. cucumerina*, and *E. nigrum* increased root hair number by 124%, 104%, 82%, and 53%, respectively, relative to the control. In contrast, VOCs from *M. bolleyi* and *C. funicola* inhibited root hair formation, decreasing the number of root hairs by 35% and 18%, respectively.

The biomass of barley seedling shoots and roots was assessed on day 12 of in vitro growth. VOCs from *C. fastigiata* demonstrated a strong stimulatory effect, increasing root biomass by 80% and shoot biomass by 50%, relative to the control (Figure 1D). *E. nigrum* exhibited a similar but reversed pattern, enhancing shoot biomass by 80% and root biomass by 50%. VOCs from *C. fumosorosea* promoted root growth more strongly than shoot growth, with increases of 44% and 15%, respectively. *P. cucumerina* VOCs produced a balanced promotive effect, increasing root and shoot biomass by 32% and 24%, respectively. In contrast, *C. funicola* and *M. bolleyi* VOCs reduced root biomass by 50% and 25%, respectively. The growth-suppressing effect of *C. funicola* also extended to the shoots, resulting in a 47% reduction.

### 3.2. The Effect of Fungi-Emitted VOCs on Red Clover Growth In Vitro

**Figure 2:**
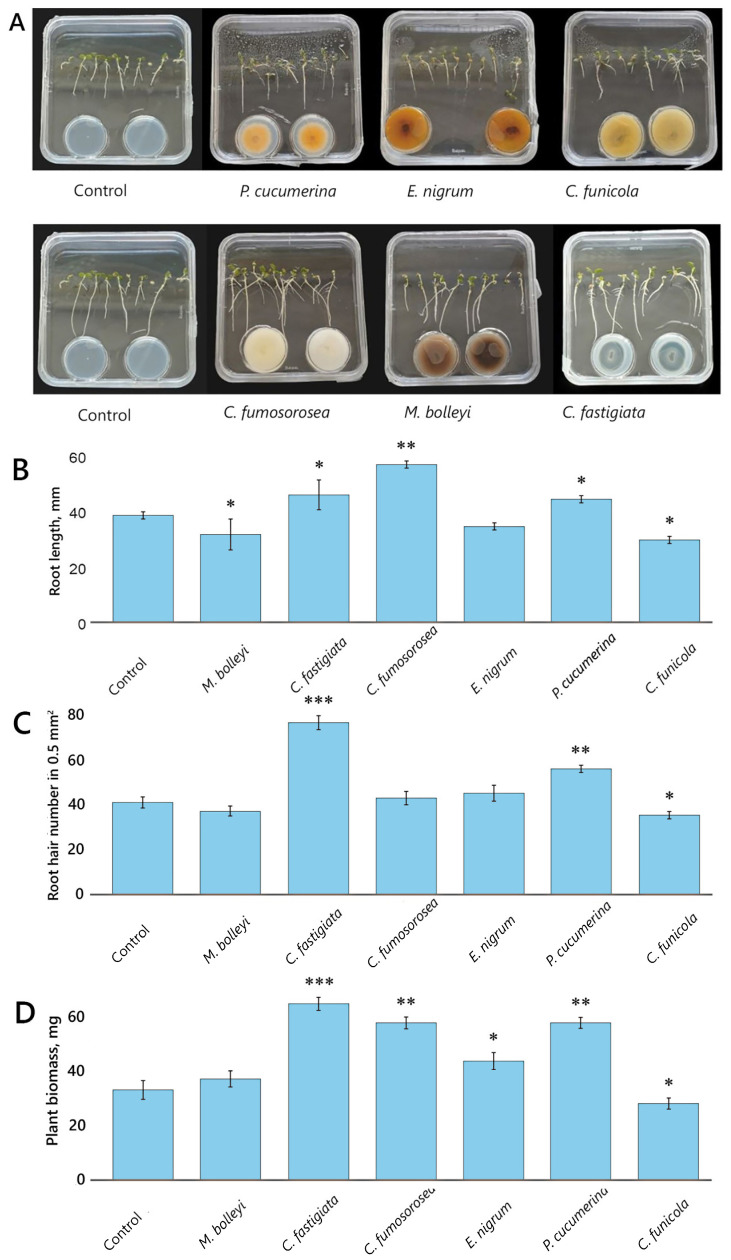
Development of red clover exposed to VOCs emitted by *P. cucumerina*, *E. nigrum*, *C. funicola*, *C. fumosorosea*, *M. bolleyi*, and *C. fastigiata*, in plate-in-plate assays. (**A**) red clover seedlings after 5 (upper panel) and 7 (bottom panel) days of growth under exposure to fungal VOCs; (**B**) root length on day 7; (**C**) number of root hairs per 0.5 mm^2^ area on day 7; (**D**) fresh seedling biomass after 12 days of growth. Notes: the sampling replicates are *n* = 50 in (**B**,**D**), and *n* = 10 in (**C**); the symbols *, **, and *** indicate significant differences relative to control without fungal VOC exposure at *p* < 0.05, *p* < 0.01, and *p* < 0.001, respectively (Student’s *t*-test).

To broaden the study and assess the responses of different plant species to endophytic fungal VOCs, red clover ‘Arimaičiai’ was included as a representative Fabaceae crop. The same six fungal isolates tested on barley were evaluated on red clover. Exposure to fungal VOCs in the closed plate-in-plate system elicited distinct growth responses in red clover seedlings, highlighting differences among the fungal isolates (Figure 2A–D).

Red clover root length, measured on day 7, was significantly increased by *C. fumosorosea* VOCs treatment, with a gain of 41% compared to the control (Figure 2B). *C. fastigiata* and *P. cucumerina* produced moderate effects, increasing root length by 18%. In contrast, *C. funicola* suppressed root growth, reducing root length by 23%, while *M. bolleyi* caused an 18% decrease. *E. nigrum* showed no significant effect.

The number of root hairs, assessed on day 7, was significantly increased by *C. fastigiata* VOCs, resulting in an 85% gain, followed by *P. cucumerina*, which induced a 39% increase (Figure 2C). *C. funicola* inhibited root hair proliferation, reducing root hair numbers by 21%. *M. bolleyi*, *C. fumosorosea*, and *E. nigrum* did not have a significant effect.

Red clover seedling biomass was assessed after 12 days of in vitro growth under VOC exposure. VOCs from *C. fastigiata*, *P. cucumerina*, and *C. fumosorosea* notably increased seedling biomass by 98%, 78%, and 78%, respectively, while exposure to *E. nigrum* VOCs resulted in a 35% increase (Figure 2D). In contrast, *C. funicola* VOC treatment reduced seedling biomass by 21%, while *M. bolleyi* had no significant effect on biomass.

**Figure 3:**
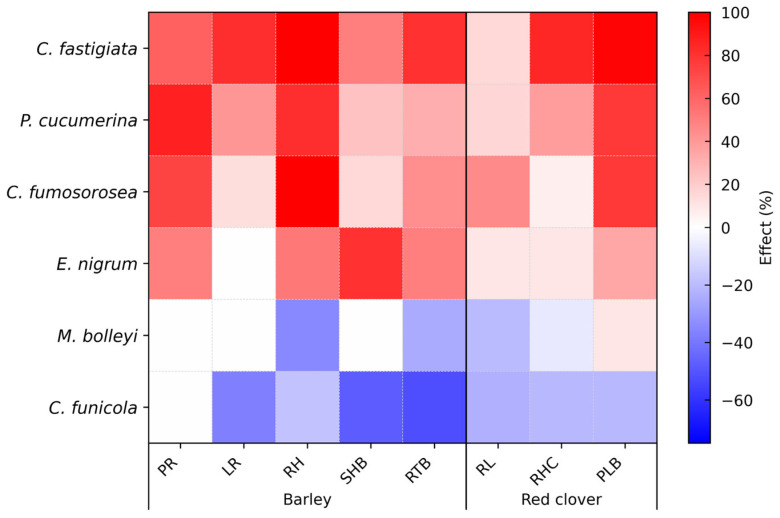
Effects of fungal VOC exposure treatments emitted by the colonies of six fungal species on barley and red clover growth parameters in a plate-in-plate experiment. Colors indicate significant changes relative to control (*p* < 0.05): red = positive, blue = suppressive, white = neutral. Notes: for barley, PR—primary root number, LR—lateral root number, RH—root hair number, SHB—shoot biomass, and RTB—root biomass; for red clover, RL—root length, RHC—root hair number, and PLB—plant biomass.

The heat map visualization reveals a clear differentiation in the effects of the six fungal VOC exposure treatments on barley and red clover early growth parameters assessed in the plate-in-plate assays (Figure 3). Among the tested fungi, exposure to VOCs from *C. fastigiata* exhibited the most consistent growth-promoting activity, stimulating seven of the eight measured traits across both plant species. *P. cucumerina* VOCs likewise showed predominantly stimulatory effects, enhancing barley root growth and red clover biomass, with moderate positive effects on the remaining traits. *C. fumosorosea* VOCs treatment markedly enhanced barley primary root and root hair growth. The effects of *E. nigrum* varied across the traits, showing a strong positive effect on barley shoot biomass and moderate stimulation of its root traits.

In contrast to the four growth-promoting fungal isolates, *C. funicola* VOC exposure had a growth-suppressing effect, markedly reducing shoot and root biomass in barley and adversely affecting other traits in both barley and red clover. VOCs from *M. bolleyi* elicited mostly neutral or negative responses, including a strong reduction in root hair formation in barley and moderate suppressive effects on other measured parameters.

Overall, the heat map provides an overview of species-specific VOC-mediated effects on early root and shoot development in barley and red clover. It highlights the strongest stimulatory effects of *C. fastigiata*, followed by *P. cucumerina*, the notable enhancement of barley primary root and root hair growth by *C. fumosorosea*, mixed responses to *E. nigrum*, and the growth-suppressing effects of *C. funicola* and *M. bolleyi*.

### 3.3. Effect of Seed Inoculation by Fungal Spore Suspensions on Spring Barley Growth

Three fungi tested in the present VOC experiment—*C. fastigiata*, *P. cucumerina*, and *P. fimeti*—were previously shown to stimulate growth in spring barley and Italian ryegrass through seed inoculation [31]. In this study, the inoculation experiment with spring barley was expanded to include three additional endophytic fungi assessed in the VOC treatment experiment: *C. fumosorosea*, *M. bolleyi*, and *C. funicola*. Including these newly tested fungi allows a broader comparison of VOC effects versus inoculation effects.

Microscopic examination with Trypan Blue staining revealed that the roots were successfully colonized by the fungus. Hyphal structures were observed in 80–90% of root samples on post-inoculation day 4, compared with approximately 30% in the inoculation-free control (root sampling *n* = 50 per treatment).

**Figure 4:**
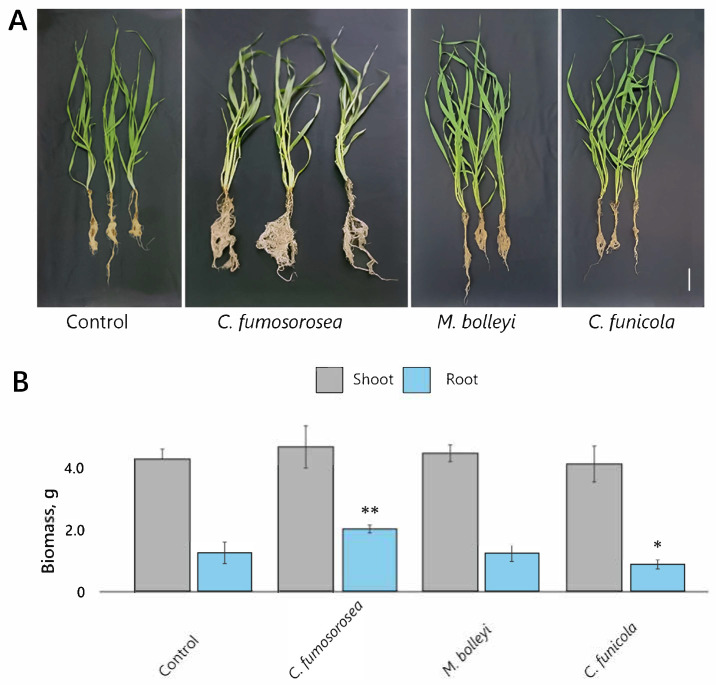
Effects of fungal inoculations on spring barley grown in multi-cavity trays in a greenhouse. (**A**) Representative images of plants following seed inoculation with *C. fumosorosea*, *M. bolleyi*, or *C. funicola*. (**B**) Fresh shoot and root biomass 30 days post-inoculation (*n* = 54 plants per treatment). Note: the symbols * and ** indicate significant differences relative to the control at *p* < 0.05 and *p* < 0.01, respectively (Student’s *t*-test); scale bar—5 cm in (**A**).

Evaluation of fresh shoot and root biomass in barley plants 30 days post-inoculation revealed distinct growth responses, reflecting variation among the fungal inoculations. While shoot biomass of inoculated plants was comparable to that of the control, root growth varied considerably. The root growth was most strongly stimulated by inoculation with *C. fumosorosea*, which increased root biomass by 67% relative to the control (Figure 4A,B). In contrast, *C. funicola* suppressed root growth, resulting in a 28% reduction. *M. bolleyi* inoculation had no measurable effect on root biomass relative to the control.

### 3.4. Distribution of VOCs Emitted by Endophytic Fungi (GC–MS Analysis)

For the VOC spectrum analysis conducted by gas chromatography–mass spectrometry (GC–MS), three fungal species were selected. *C. fastigiata* and *P. cucumerina* were chosen because of their strong growth-promoting effects observed in the current study and in the previous experiment [31]. *P. fimeti* and a three-fungal mix were selected because both showed growth-promoting effects in barley and Italian ryegrass inoculation assays comparable to *C. fastigiata* and *P. cucumerina* [31]. It is important to note that *P. fimeti* was successfully included in inoculation experiments [31], but it was excluded from the present VOC exposure assays due to its strong airborne spore dispersal, which leads to direct detrimental seedling–mycelium contact.

**Figure 5:**
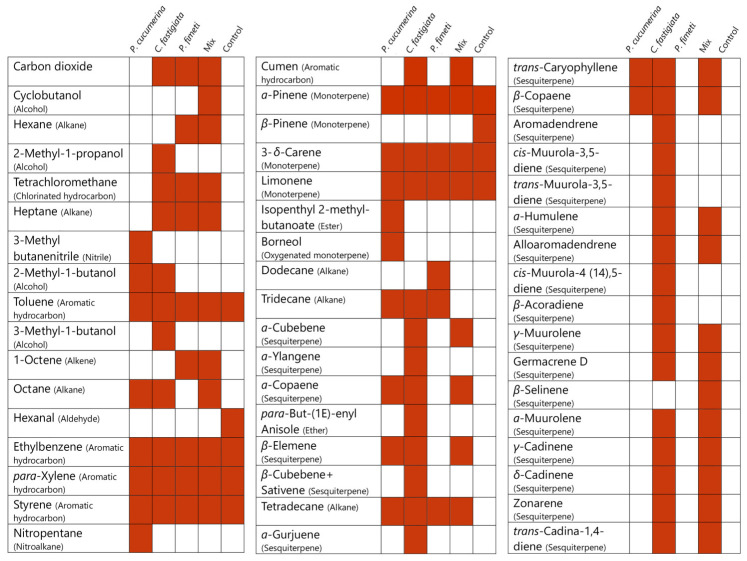
Volatile organic compounds (VOCs) profiles emitted by the colonies of *C. fastigiata*, *P. cucumerina*, and *P. fimeti*, and a mixed three-fungal sample, collected from the colonies grown on PDA medium in Petri dishes and analyzed by GC–MS; Control: PDA medium only. The additional VOCs profile data are provided in Supplementary Table S1 and Figure S1.

Five samples were analyzed by GC–MS for VOS emission: mycelial colonies of *C. fastigiata*, *P. cucumerina*, and *P. fimeti*; a three-fungal mix sample; and control Petri dishes containing only growth medium. Each sample type was analyzed in triplicate (*n* = 3) with two technical repetitions. The distribution of VOC obtained from three fungal isolates and their mixture is shown in Figure 5, with detailed profiles provided in Table S1 and Figure S1. In total, 48 distinct VOCs were identified in the fungal samples. Nine additional compounds, which were also present in the control Petri dishes with PDA medium, have been excluded from this count.

*C. fastigiata*, which showed the strongest growth-promoting activity in the plate-in-plate assays, produced the highest number of VOCs (33, excluding compounds present in the control), dominated by 22 sesquiterpene compounds (Figure 5, Figures S1 and S2, Table S1). Its VOC profile also dominated the three-fungal mix sample. From *P. cucumerina*, the most abundant VOCs were sesquiterpenes (4 compounds) and alkanes (3 compounds). Four sesquiterpenes—*α*-copaene, *β*-copaene, *β*-elemene, and *trans*-caryophyllene—identified in *P. cucumerina* were also present in *C. fastigiata*. These results suggest that the growth-stimulating effects of *C. fastigiata* and *P. cucumerina* may be closely linked to their production of bioactive terpenes. No sesquiterpenes were detected in *P. fimeti* or in the control Petri dishes.

## 4. Discussion

This study demonstrates that endophytic fungi isolated from grass roots produce species-specific volatile organic compounds (VOCs) with distinct effects on the early development of spring barley (*Hordeum vulgare*) and red clover (*Trifolium pratense*). The contrasting growth responses observed among the six isolates highlight the functional diversity of fungal endophytes and are consistent with previous studies, which demonstrate endophyte–host interactions ranging from mutualistic to neutral or inhibitory [1,3,6,37,38]. Our findings demonstrate that specific fungal VOCs can predict systemic growth responses and developmental outcomes across phylogenetically and physiologically distant plants. Furthermore, this study provides a framework for identifying fungal isolates with potential benefits for both cereals and forage legumes.

### 4.1. Strong Growth-Promoting Effects of C. fastigiata and P. cucumerina

Among the tested fungi, *C. fastigiata* and *P. cucumerina* VOC treatments exhibited the most consistent growth-promoting activity, enhancing multiple root traits and seedling biomass in both species. These results are consistent with our previous inoculation studies, which demonstrated improved growth of barley and Italian ryegrass, with stronger effects on roots than on shoots [31]. Moreover, *Cadophora* spp. have been reported to increase tomato biomass [39], stimulate root development in perennial ryegrass [40,41], and suppress *Fusarium* wilt in *Cucumis melo* [42]. Together, these findings highlight the dual functionality of *Cadophora* spp. as both biostimulants and biocontrol agents.

Although *P. cucumerina* is widely known as a pathogen of Cucurbitaceae and other vegetable crops [43,44], no infections have been reported in Poaceae. Beyond its pathogenicity, several studies have highlighted its potential as a biological control agent; for example, it exhibits nematophagous activity against potato cyst nematodes [45] and has been explored as a bioherbicide for managing *Cirsium arvense* [46]. Our previous seed inoculation study [31] and the current VOC exposure experiment suggest that *P. cucumerina* may act as a growth promoter in grasses. However, its pathogenicity toward cucurbits and other crops may limit its agronomic application.

### 4.2. Moderate and Trait-Specific Effects of C. fumosorosea and E. nigrum

*C. fumosorosea* notably enhanced the root growth in both barley and red clover. Although traditionally studied as an entomopathogen [47,48], endophytic strains have recently been shown to promote plant growth, as in eggplant [33]. Our findings support this dual ecological role, suggesting that grass root-associated *Cordyceps* isolates may emit growth-activating VOCs and modulate plant metabolism in ways that favor root expansion.

In contrast, *E. nigrum* moderately stimulated several traits in barley and red clover. Its capacity to produce antifungal metabolites and promote root growth, as previously documented in sugarcane [32], suggests that this species can act as a moderate growth promoter; however, such effects appear to be host- and condition-dependent.

### 4.3. Growth-Suppressing and Neutral Responses from C. funicola and M. bolleyi

In contrast to the growth-promoting effects described above, VOC treatment from *C. funicola* exerted a strongly adverse effect, suppressing root and shoot growth of barley and red clover seedlings. *Chaetomium* species are common endophytes that often colonize Poaceae roots [36,49] and can thrive in harsh environments such as coastal dunes and the semi-arid Caatinga [50,51]. Notably, while other *Chaetomium* species are widely used in the production of commercial biofungicides or biostimulants [52,53], *C. funicola* is not included among them. Comparing the VOC profiles of beneficial *Chaetomium* species, such as *C. globosum*, with those of phytotoxic *C. funicola* strains may help clarify the factors underlying their contrasting effects.

In our study, *M. bolleyi* exhibited neutral to weakly inhibitory effects. VOC emissions suppressed root hair proliferation in barley and red clover, suggesting that *M. bolleyi* may produce metabolites that reduce root vigor. This was unexpected, as *M. bolleyi* was found to be the most common root endophyte across *Lolium* and *Festuca* species [34] and was therefore presumed to benefit plant growth. Overall, its functional role appears variable, depending on host and environmental conditions [5,35,54]; for example, in *Brachypodium distachyon* (Poaceae), it typically establishes asymptomatic, neutral interactions [55].

### 4.4. Inoculation Performance Reflects VOC Exposure Trends

Importantly, the trends observed in VOC exposure experiments closely matched those in inoculation trials. Barley seed inoculation with *C. fumosorosea* increased root biomass, *M. bolleyi* remained neutral, and *C. funicola* reduced growth, which was consistent with outcomes from the VOC treatment. In addition, comparison of the current VOC-based treatments with previous inoculation studies on *C. fastigiata* and *P. cucumerina* [31] reinforces the notion that these two fungi act as strong biostimulants. It also shows that in vitro assessment of VOC-mediated growth-promoting effects offers a rapid and reliable method for screening fungal isolates. A similar alignment between VOC profiles and inoculation performance has been reported for *Metarhizium anisopliae*, with effects on the growth of *Arabidopsis*, tomato, and maize [56].

### 4.5. VOC Profiles Reflect Differentiation Among Endophytes in Growth Promotion

VOCs can influence plant development at multiple levels, enhancing both growth and defense responses [57,58,59,60,61]. VOC production is often taxonomically constrained [10,16,62], with terpenes—especially sesquiterpenes—being linked to growth promotion and immunity [63].

In our study, GC–MS profiling provided insights into potential links between the VOC composition and the resulting plant responses. The top growth-promoting isolate *C. fastigiata* emitted the richest sesquiterpene (SQT) blend (22 compounds) and was associated with the strongest increases in root growth, root hair proliferation, and overall biomass accumulation in both barley and red clover. Four sesquiterpenes—*α*-copaene, *β*-copaene, *β*-elemene, and *trans*-caryophyllene—were detected in both *C. fastigiata* and *P. cucumerina* profiles. The similarity in responses observed for these two species suggests that sesquiterpene-rich VOC mixtures may confer broad-spectrum growth-promoting effects across multiple hosts. Evidence from other studies supports this pattern; for instance, SQTs from *Laccaria bicolor*, including (–)-thujopsene, enhance lateral root formation in *Populus* and *Arabidopsis* [12]. Although our study did not investigate the underlying mechanisms, previous studies suggest that VOCs, such as cedrene from *Trichoderma guizhouense* [64] and 1-octen-3-ol or 2-heptylfuran [59], can promote auxin-dependent root growth, which is consistent with the pronounced root growth enhancement observed in our study. To better assess the functional effects of VOCs observed in this study, future research using functional assays—such as testing individual VOCs or synthetic blends, employing mutant or knock-out strains, and conducting dose–response experiments—may help establish direct links between VOCs and plant growth responses.

## 5. Conclusions

Our research demonstrates that grass-root-associated fungal endophytes produce species-specific VOCs that specifically affect early development in barley and red clover. VOC exposure plate-in-plate assays revealed clear, predictive trends in growth-promoting effects, which were supported by inoculation outcomes. Among the six grass-root-derived fungal isolates, *Cadophora fastigiata* was the most effective growth promoter, significantly increasing plant biomass and enhancing root development in two contrasting crops, barley and red clover. Furthermore, *C. fastigiata* exhibited a distinctive VOC profile dominated by 22 sesquiterpenes, suggesting a potential stimulatory role for these compounds. Overall, our results highlight the utility of in vitro VOC exposure assays as a rapid screening tool for selecting efficient fungal isolates. Together, these findings pave the way for exploiting VOC-producing endophytes as innovative, eco-friendly tools to improve crop growth and resilience. Despite these promising results, limitations remain, including the need for broader crop testing to confirm the spectrum of beneficial effects, to validate these effects under field conditions, and to further investigate the functional roles of individual VOCs and their blends in plant metabolism.

## Data Availability

The original contributions presented in this study are included in the article/Supplementary Material. Further inquiries can be directed to the corresponding author.

## Supplementary Materials

The following supporting information can be downloaded at: https://www.mdpi.com/article/10.3390/microorganisms14030533/s1, Table S1: Fungal VOC composition spectra; Figure S1: Typical total ion chromatogram (TIC). Figure S2: View of VOC sampling. Ref. [65] can be found in Supplementary Materials.
