# Supplementary material for: Contrasting Effects of Grass-Derived Endophytic Fungal VOCs on Early Growth of Spring Barley and Red Clover: From Stimulation to Suppression
Source: Microorganisms. 2026 Feb 25;14(3):533. doi: 10.3390/microorganisms14030533 (PMC13029737; doi:10.3390/microorganisms14030533)
Supplement: Supplementary file 1 [file microorganisms-14-00533-s001.zip › Table S1 Fungal VOC compostion spectra.pdf]

Table S1. VOC composition of mycelial colonies of *C. fastigiata*, *P. cucumerina*, and *P. fimeti*; a mixed sample of all three fungi; and control Petri dishes With PDA growth medium. Each biological repetition (n = 3) of the sample type had two technical replicates

RT	Compound (Chemical group)	$RI_{Lit/Exp}$	Amount intervals (n=6), %				
			Plectospaerella cucumerina	*Cadophora fastigiata*	*Paraphoma femiti*	Three fungal mix	Growing medium
1.492	Carbon dioxide	-		0.80 - 1.11	0.2 - 1.1	1.2 - 2.5	
1.758	Cyclobutanol	-/567				1.84 - 3.39	
2.103	Hexane	600/600			6.91 - 7.24	3.56 - 5.82	
2.339	2-Methyl-1-propanol	622/621		2.22 - 2.39			
2.602	Tetrachloromethane	661/661		0.06 - 0.22	0.1 - 0.37	tr. - 0.17	
2.991	Heptane	700/700		tr. - 0.07	tr.	tr. - 0.19	
3.484	3-Methyl butanenitrile	730/730	0.98 - 1.36				
3.614	2-Methyl-1-butanol	732/732	2.08 - 2.12	0.51 - 0.99			
4.027	Toluene*	759/760	0.88 - 0.96	tr. - 0.58	0.26 - 0.78	0.15 - 0.54	1.33- 2.04
4.461	3-Methyl-1-butanol	763/760		tr. - 0.16			
4.463	1-Octene	794/794			tr. - 0.41	0.73 - 1.23	
4.639	Octane	800/800	0.31 - 0.49	0.11 - 0.37		0.37 - 0.5	
4.769	Hexanal*	802/802					0.29 - 0.42
5.91	Ethylbenzene*	864/864	2.1 - 2.7	0.42 - 0.63	0.84 - 1.05	0.39 - 0.67	2.04 - 2.15
6.108	*para*-Xylene*	870/869	4.99 - 5.22	1.53 - 1.89	2.52 - 3.89	1.95 - 2.36	6.03 - 6.76
6.578	Styrene*	890/890	9.00 - 9.28	10.14 - 12.66	16.34 - 18.19	12.00 - 15.19	19.82 - 23.7
6.867	Nitropentane	925/926	tr. - 1.84				
7.311	Cumene	930/930		0.06 - 0.11		0.05 - 0.11	
7.553	α-Pinene*	939/939	0.39 - 0.46	0.11 - 0.27	0.29 - 0.48	0.09 - 0.28	0.89 - 1.13
8.57	β-Pinene*	979/979					0.13 - 0.42
9.32	3-δ-Carene*	1009/1009	0.27 - 0.44	0.15 - 0.28	0.20 - 0.28	tr. - 0.18	0.94 - 1.17
9.795	Limonene*	1029/1030	1.19 - 1.39	0.51 - 0.66	0.36 - 0.45	0.29 - 0.47	1.11 - 1.87
11.697	Isopenthyl 2-methylbutanoate	1101/1102	tr. - 0.15				

13.393	Borneol	1169/1170	tr. - 0.15				
15.134	Dodecane	1200/1200			tr. - 0.1		
16.058	Tridecane	1300/1300	0.05 - 0.1	0.06 - 0.1	0.10 - 0.11		
16.569	*α*-Cubebene	1348/1348		0.05 - 0.09		tr.	
17.023	*α*-Ylangene	1375/1375		0.12 - 0.17			
17.166	*α*-Copaene	1376/1376	tr.	0.52 - 0.7		0.11 - 0.18	
17.277	*para*-But-(1*E*)-enyl Anisole	1380/1381		0.08 - 0.11			
17.289	*β*-Cubebene	1389/1389				tr.	
17.411	*β*-Elemene	1390/1390	0.17 - 0.26	0.07 - 0.1		tr. - 0.05	
17.617	Tetradecane	1400/1400	0.25 - 0.32	0.05 - 0.09	0.29 - 0.34	0.09 - 0.11	
17.739	*α*-Gurjunene	1410/1409		tr.			
19.999	*trans*-Caryophyllene	1419/1420	0.35 - 0.44	7.34 - 7.88		3.22 - 3.5	
18.192	*β*-Copaene	1432/1433	0.11 - 0.16	0.43 - 0.54		0.18 - 0.23	
18.481	Aromadendrene	1440/1440	0.25 - 0.33	tr.			
18.458	*cis*-Muurola-3,5-diene	1450/1450		tr.			
18.515	*trans*-Muurola-3,5-diene	1452/1452		0.09 - 0.11			
18.642	*α*-Humulene	1454/1454		tr.		tr.	
18.718	Alloaromadendrene	1460/1460		4.11 - 4.31		0.54 - 0.79	
18.958	*cis*-Muurola-4(14),5-diene	1466/1466		0.05 - 0.09			
18.967	*β*-Acoradiene	1472/1473		0.07 - 0.1			
19.094	*γ*-Muurolene	1478/1478		2.54 - 3.73		tr. - 0.06	
19.283	Germacrene D	1482/1485		0.11 - 0.18		0.51 - 0.58	
19.299	*β*-Selinene	1490/1490				tr. - 0.08	
19.386	*α*-Muurolene	1500/1501		2.92 - 3.69		0.59 - 0.78	
19.661	*γ*-Cadinene	1513/1513		0.07 - 0.11		tr.	
19.743	*δ*-Cadinene	1523/1523		0.08 - 0.14		tr.	
19.824	Zonarene	1537/1536		0.31 - 0.46		tr. - 0.07	
19.947	*trans*-Cadina-1,4-diene	1538/1538		0.17 - 0.21			
	**Total number of identified VOCs**		**20**	**40**	**15**	**31**	**9**
	Total number of alkanes		3	3	3	2	0

	Total number of monoterpenes		3	3	3	3	4
	Total number of sesquiterpenes		4	22	0	15	0
	Total number of alcohols		1	3	0	0	0

RT-retention time, min; $RI_{Lit/Exp}$-the retention indices taken from literature (webbook.nist.gov and Adams, R P., 2001) and those determined experimentally relative to the retention times of a series of *n*-alkanes with (C_7–C_{30}) linear interpolation; tr.—trace amounts < 0.05 %, *— compounds associated with growth medium and Petri dish.

Adams, R.P. *Essential Oil Components by Quadrupole Gas Chromatography/Mass Spectrometry*, 3rd ed.; Allured Publishing Corp.: Carol Stream, IL, USA, 2001.
