# Supplementary material for: Contrasting Effects of Grass-Derived Endophytic Fungal VOCs on Early Growth of Spring Barley and Red Clover: From Stimulation to Suppression
Source: Microorganisms. 2026 Feb 25;14(3):533. doi: 10.3390/microorganisms14030533 (PMC13029737; doi:10.3390/microorganisms14030533)
Supplement: Supplementary file 1 [file microorganisms-14-00533-s001.zip › Figure S1 Total ion chromatogam of C. fastigiate VOCs.pdf]

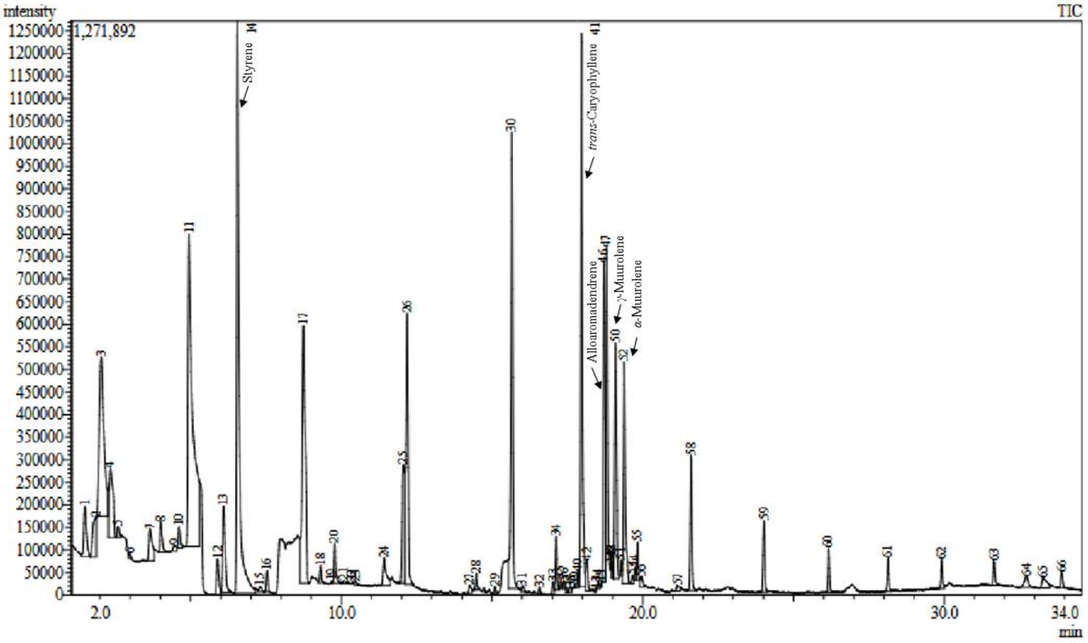

Figure S1. Typical total ion chromatogram (TIC) of VOCs emitted by *Cadophora fastigiata* mycelium grown on PDA medium. Styrene as an impurity from the Petri dish and the most abundant sesquiterpenes are marked in the chromatogram with corresponding peaks. All other identified compounds and their relative amounts are presented in the Table S1. Peaks indicated by numbers 3, 11, 17, 25, 26, 30, 47, 58 and so on are siloxane derived compounds associated with SPME fiber.
