# Supplementary material for: Contrasting Effects of Grass-Derived Endophytic Fungal VOCs on Early Growth of Spring Barley and Red Clover: From Stimulation to Suppression
Source: Microorganisms. 2026 Feb 25;14(3):533. doi: 10.3390/microorganisms14030533 (PMC13029737; doi:10.3390/microorganisms14030533)
Supplement: Supplementary file 1 [file microorganisms-14-00533-s001.zip › Figure S2 View of VOC sampling.pdf]

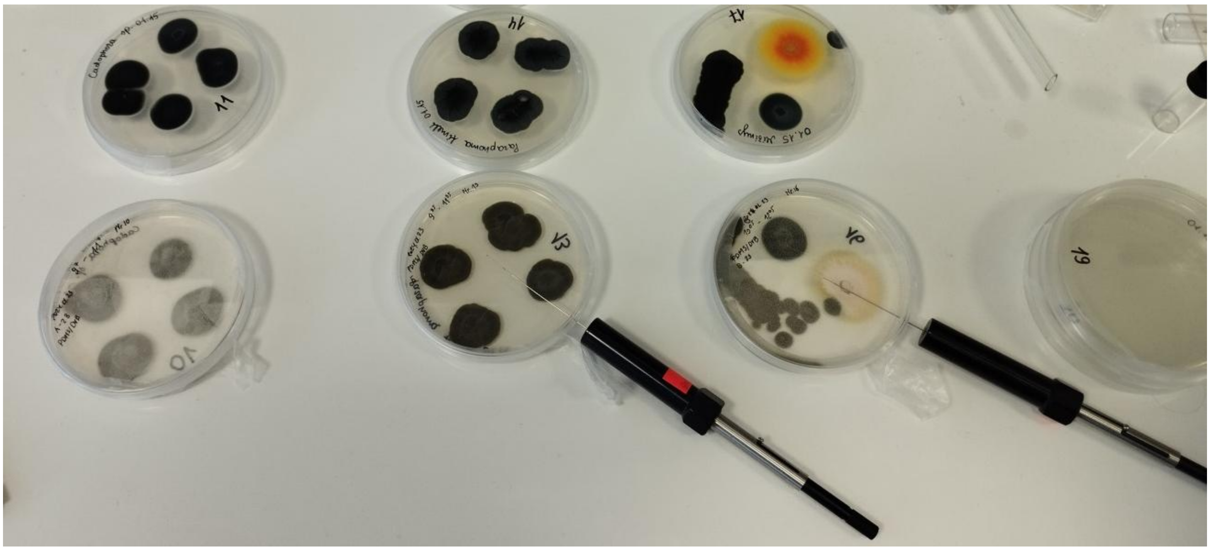

Figure S2. The representative view in the figure illustrates VOC sampling for *P. fimeti* and mixed samples of *C. fastigiata*, *P. cucumerina*, and *P. fimeti*.
